# Stem Cells for Cartilage Regeneration: A Roadmap to the Clinic

**DOI:** 10.1155/2018/7348560

**Published:** 2018-04-11

**Authors:** Celeste Scotti, Alberto Gobbi, Norimasa Nakamura, Giuseppe M. Peretti

**Affiliations:** ^1^Novartis Institutes for Biomedical Research, Novartis Pharma AG, 4056 Basel, Switzerland; ^2^Orthopaedic Arthroscopic Surgery International (OASI) Bioresearch Foundation, Gobbi Onlus, Milan, Italy; ^3^Institute for Medical Science in Sports, Osaka Health Science University, Osaka, Japan; ^4^Center for Advanced Medical Engineering and Informatics, Osaka University, Osaka, Japan; ^5^IRCCS Istituto Ortopedico Galeazzi, 20161 Milan, Italy; ^6^Department of Biomedical Sciences for Health, University of Milan, Milan, Italy

Cell therapies for cartilage repair date back to 1987 when the first autologous chondrocyte implantation (ACI) procedure was performed. Since then, more than 30,000 patients have been treated with these techniques, which should not be considered “experimental” anymore. Over the years, several technical improvements have been implemented, leading to incremental advances in the surgical procedure, as reviewed by Y. Nam et al. However, a predictable, standardized, and durable regeneration of hyaline cartilage tissue, capable to withstand the mechanical forces acting in the joint and to prevent joint degeneration, remains an unmet medical need [1].

In this special issue entitled “Stem Cells for Cartilage Regeneration: A Roadmap to the Clinic,” the authors addressed several relevant topics, ranging from advanced in vitro and in vivo models to alternative cell sources, including induced pluripotent stem cells (iPS), and from smart materials to additional target tissues with high unmet medical need, such as the trachea or the temporomandibular joint (TMJ).

A key bottleneck to improved therapies is represented by reliable in vitro and in vivo models, capable to predict the clinical outcome. Strong advances have been made in this field, towards the development of high-throughput systems that allows testing multiple conditions with reproducible, quick, and affordable methods, and S. Lopa et al. provided a comprehensive review of microfluidics and bioprinting applications. Another important topic is quality control in cell therapies, in order to better standardize the clinical outcome. K. Shiraishi et al. reported an interesting study analysis of mRNA and miRNA correlated with in vivo cartilage repair, which may open new avenues for patient stratification and selection, beyond the mere quality control. Regarding in vivo models, a translational model capable to duplicate the challenging clinical scenarios has yet to be developed. M. Lo Monaco et al. reviewed extensively this topic, ranging from small to large animal models and providing critical insights for study planning.

The use of articular chondrocytes as a cell source has been considered a bottleneck to a more robust and reproducible regeneration of the articular surface, because of their typical age-dependency and interdonor variability in the cartilage-forming capacity [2]. For this reason, recent research focused on alternative cell sources and experimental models in order to overcome the intrinsic limitations of autologous cell therapies based on articular chondrocytes. J. N. Fisher et al. reviewed recent advances in preclinical and clinical research on a number of tissue sources of progenitor cells for cartilage repair, highlighting pros and cons of each of them, with a focus on the potential for clinical translation. K. D. Jorgenson et al. presented a suspension bioreactor incorporating microcarrier technology for the efficient culture of synovial fluid-derived MSCs, which can potentially support further research with this cell source. Infrapatellar fat pad-derived cells gained attention because of their easy accessibility and chondrogenic potential. J. F. C. do Amaral et al. reviewed the potential of infrapatellar fat pad cells, discussing their potential for cartilage repair and the ontogeny relationship with other joint-derived cells and concluding with some perspective for translational trials using this cell source. Another cell type that showed promising preclinical data, with also a clinical trial ongoing, is synovial MSC [3]. Y. Ikeda et al. reported a successful approach to improve further the chondrogenic activity of synovial MSC, without the upregulation of hypertrophic and osteogenic genes, by enhanced IGF-1 expression. Last, human-induced pluripotent stem cells (hiPSCs) gained a lot of attention in the last decade, representing a new hope for several life-threatening and incurable diseases. Y. A. Rim et al. reported a relevant analysis of the chondrogenic potential among hiPSCs from different tissues: the finding that cord blood mononuclear cells represent a better source may support further research in this direction.

Biomaterials are a mainstay of regenerative medicine, especially for articular cartilage. However, it is still a matter of controversy whether a scaffold is strictly needed or not. In this special issue, both approaches are reported. Interestingly, F. Hached et al. reported the positive impact of a polysaccharide hydrogel on encapsulated MSCs, with respect to cell viability and ability to secrete potentially therapeutic factors. Regarding scaffold-free approaches, M. P. Stuart et al. reported a valuable method to engineer spheroids by using a micromolded nonadhesive hydrogel, without the use of growth factors.

In this special issue, the authors addressed a series of topics of relevance for the successful translation of preclinical approaches. Cell sources, biomaterials, animal models, and cell manufacturing are all critical factors for cartilage repair, which require additional work to pave the way to the next generation of regenerative therapies, possibly capable to restore durably both joint surface and function in patients in need.

*Celeste Scotti* *Alberto Gobbi* *Norimasa Nakamura* *Giuseppe M. Peretti*
